# Genetic characterization of *Plasmodium vivax* isolates from Pakistan using circumsporozoite protein (*pvcsp)* and merozoite surface protein-1 (*pvmsp-1*) genes as genetic markers

**DOI:** 10.1186/s12936-021-03654-w

**Published:** 2021-02-25

**Authors:** Zainab Bibi, Anam Fatima, Rehana Rani, Ayesha Maqbool, Samea Khan, Shumaila Naz, Shahid Waseem

**Affiliations:** 1 Department of Molecular Biology, Virtual University of Pakistan, Lahore, Pakistan; 2 Department of Medicine, Polyclinic Hospital, Islamabad, Pakistan; 3 Department of Life Sciences, Abasyn University, Islamabad, Pakistan; 4 Department of Biological Sciences, National University of Medical Sciences, Rawalpindi, Pakistan; 5 Alpha Genomics (Pvt) Ltd, Islamabad, Pakistan; 6 ABO SCIENTIFIC, Dhamial Road, Rawalpindi, Pakistan

**Keywords:** *Plasmodium vivax*, Malaria, Genetic diversity, CSP, MSP-1

## Abstract

**Background:**

*Plasmodium vivax* contributes to over 70% malaria burden in Pakistan, but limited data exists on various aspects including genetic diversity of the parasite as compared to other parts of the world. Since the information about the genetic diversity of *P. vivax* assists to understand the population dynamics of the parasite, the current study was designed to understand population divergence of *P. vivax* in Pakistan using circumsporozoite protein *(pvcsp)* and merozoite surface protein-1 *(pvmsp-1)* genes as molecular markers.

**Methods:**

The PCR for *pvcsp* and *pvmsp-1* genes was carried out for 150 *P. vivax* isolates, followed by DNA sequencing of 35 and 30, respectively. Genetic diversity and polymorphism were analysed using ChromasPro, ClustalW, MEGA7, DnaSP v.5 and WebLogo programs.

**Results:**

The PCR for *pvcsp* and *pvmsp-1* genes was carried out for 150 *P. vivax* isolates and resulting the PCR products of 1100 bp for *pvcsp* and ~ 400 bp for *pvmsp-1* genes, respectively. In the central-repeat region (CRR) of *pvcsp* gene, sequences comprised of four variable repeats of PRMs, out of which GDRADGQPA (PRM1), GDRAAGQPA (PRM2) were more extensively dispersed among the *P. vivax* isolates. Partial sequences (~ 400 bp) of block 2 of *pvmsp-1* gene depicted high level of diversity.

**Conclusion:**

The results revealed the polymorphism and genetic diversity especially at the CRR of *pvcsp* and block 2 of *pvmsp-1* genes, respectively. The base-line data presented here warrants future studies to investigate more into the genetic diversity of *P. vivax* with large sample size from across the country for better understanding of population dynamics of *P. vivax* that will help to control malaria at individual and community level.

## Background

Malaria remains a serious infectious disease of public health importance and one of the leading causes of morbidity and mortality worldwide [1]. Globally, around 42% population is at risk with almost 228 million cases of malaria with 0.4 million reported deaths in 2018 based on the data collected from 91 countries [2, 3]. Among the five *Plasmodium* species, *Plasmodium vivax* contributes around 70% malaria cases in Pakistan [4] with variable severity [5–7]. To circumvent the parasite load, there is a need to investigate the population structure, genetic diversity [8], parasite typing of the local *P. vivax* species, pattern of antigenic variations and drug resistance [9, 10]. It would lead to interruption of transmission cycle of the parasite in human host in endemic areas. Despite multiple academic research ventures, scanty data is available regarding the diverse genetic make-up of *P. vivax* in Pakistan [5, 6].

Genetic sequences of *pvcsp* and *pvmsp-1* are being used to understand the genetic diversity [11–13]. The *pvcsp* is a highly immunogenic sporozoite surface protein, hence a good vaccine candidate, is encoded by single copy gene [8] and comprises of a central repeat domain that varies across *Plasmodium* species having two non-repetitive domains at N- and C-terminals [14, 15]. The varying degree of number of peptides in the central repeat region reveals three variants of *P. vivax* namely VK210, VK247, *P. vivax*-like [16, 17]. Across the globe, these variants exhibit certain spatial predispositions. With a GDRA(A/D)GQPA amino acid repetition, VK210 strain dominates in the endemic region [18, 19]. Originating in Thailand, the VK247 strain is mostly reported from the areas where mixed infections are prominent [20]. VK247 depicts ANGAGNQPG amino acid repeat in the central region [20, 21]. In *pvcsp*, polymorphism is reported to be limited in central tandem repeat among the isolates from the regions of sub-Saharan Africa, China, Sri Lanka, Sudan and India [8–10, 13, 14, 22]. The *pvmsp-1* is expressed on the surface of the blood-stage parasite [23], a large gene, covers conserved and polymorphic regions [24] and has mosaic organization with 13 regions of variable blocks [25]. The three main regions of sequence divergence are block 2 (F1 region), 6–8 (F2 region) and 10 (F3 region) [26]. In the representative blocks, the genetically distinct *pvmsp-1* populations within the regions and polymorphism can be detected through PCR [5, 27]. Selective pressure of the host immune maintains the diversity of *pvmsp-1* gene, however, immunogenic properties can be affected by single-point mutation [28, 29]. Despite *P. vivax* contributing to 88% of malaria burden in Pakistan [5–7], data regarding genetic diversity of this key circulating species is lacking. The present study was designed to investigate the genetic diversity of *P. vivax* in Potohar region of Pakistan exploiting *pvcsp* and *pvmsp-1* genes as molecular markers. The understanding of sequence diversity in *pvcsp* would contribute to comprehend the population dynamics and transmission patten of the parasite in this region.

## Methods

### Study area

**Fig. 1:**
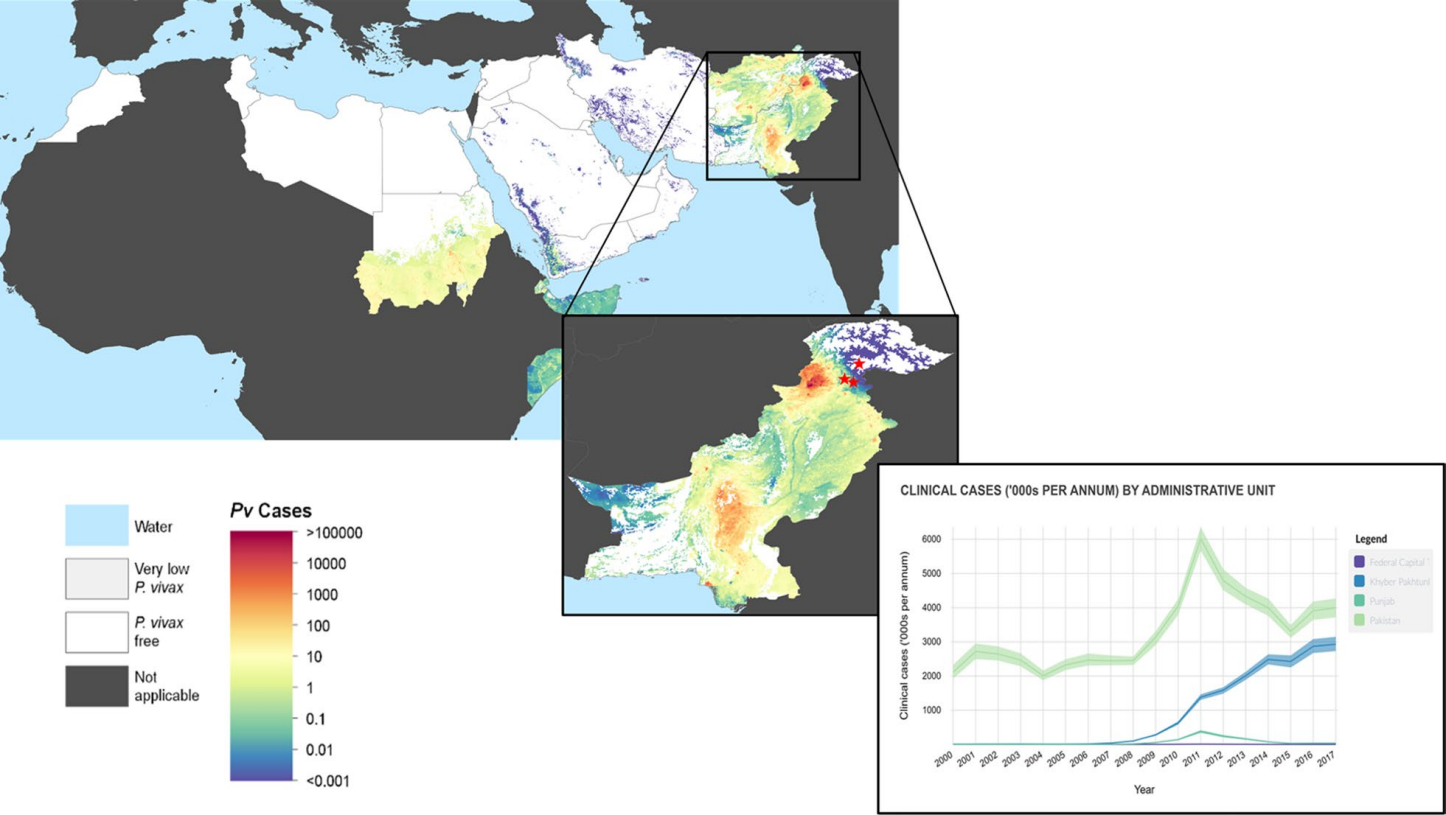
Map of *P. vivax* prevalence in EMRO region and Pakistan. The map showing all age clinical cases of *P. vivax* from year 2000–2017 of EMRO region and Pakistan. These maps were originally created by the Malaria Atlas Project, University of Oxford available at https://malariaatlas.org/. The estimated sites of the study sites described here are showed with red star: Rawalpindi and Islamabad

The study was conducted in Islamabad and Rawalpindi districts of the Punjab with longitudes 72°45′ and 73°30′ E and latitudes 33°30′ and 33°50′ N [30] (Fig. 1). The climate in the study region ranges from showery warm to chilly dry wintry with the attributes of the semi-arid region of Pakistan. The monsoon rains typically start in June, get peak in August, and finish by September. The rainfall is between 620 and 1200 mm per year. The weather and geographical settings of this region are favorable for the mosquito breeding with highest frequency reported in Rawalpindi (25.5%) and lowest in Chakwal (15.9%) [31].

### Sample collection

Blood samples (n = 150) were collected during malaria transmission season (April and October, 2019) from Pakistan Institute of Medical Sciences (PIMS), Islamabad and Rawalpindi General Hospital, Rawalpindi, Pakistan. Venous blood (5 mL) was collected in ethylene diamine tetra-acetic acid (EDTA) (BD, USA) vacutainers from malaria patients. The samples were collected from the patients fulfilling the inclusion criteria of clinical signs and symptoms (fever, chills, headache, sweats, fatigue, nausea and vomiting). Initially malaria patients were screened by microscopic examination of Giemsa-stained thin and thick blood smears to ensure the *P. vivax* parasites and exclude samples with presence of mixed *Plasmodium* species infections (mixed *P. vivax* and *P. falciparum*). Blood samples were stored at − 20 °C for a month until further analysis was carried out.

### DNA extraction and PCR amplification of *pvcsp* and *pvmsp-1* genes

Genomic DNA from 150 malarial blood samples were extracted by using standard phenol–chloroform method [32]. PCR primers for *pvcsp* and *pvmsp-1* genes were designed at Geneious software by using reference sequence of *P. vivax* (AB539044 and GQ890906). The primers for *pvcsp* gene were F: 5′-GGCCATAAATTTAAATGGAG-3′and R: 5′-ATGCTAGGACTAACAATATG-3′. The PCR conditions were as follows: pre-denaturation at 94 °C for 10 min followed by 35 cycles of annealing at 52 °C for1 min and extension at 72 °C for 1 min, followed by final extension at 72 °C for 10 min. The PCR of *pvmsp-1* gene was performed with primers F: 5′-ACATCATTAAGGACCCATACAAG 3′ and R: 5′ GCAATTTCTTTACAGTGATCTCG-3′ with similar PCR cycling conditions except that the annealing was at 56 °C for 1 min. The PCR reactions were carried out in a 25 μL reaction mixture comprising of 2μL DNA template, 0.5 mM dNTPs, 1X PCR reaction buffer (SolarBio Life Sciences, China), 0.2 mM of each primer (BGI, China), 2.5 mM MgCl_2_ and 1 unit of *Taq* DNA polymerase (BLIRT, Poland). The PCR products were visualized using 1% agarose gel (ThermoFisher, USA) stained with ethidium bromide (SolarBio Life Sciences, China) and visualized under UV-trans illuminator (ThermoFisher, USA).

### Sanger sequencing and analysis

All 150 malaria amplified genomic DNA samples showed the band size of 1100 bp for *pvcsp* gene and ~ 400 bp for partial sequence of *pvmsp-1* gene. As no variation existed in band sizes of 150 malaria samples for both genes, only 35 and 30 amplified PCR products of both *pvcsp* and *pvmsp-1* genes were selected randomly and purified, respectively by QIAquick PCR Purification Kit (Qiagen, Germany according to manufacturer’s protocol). These purified samples were then sent to Beijing Genomics Institute (BGI), China for Sanger sequencing. DNA sequences of both *pvcsp* and *pvmsp-1* genes were read and assembled on both upstream and downstream ends for 35 sequences of *pvcsp* gene and 30 sequences of *pvmsp-1* gene. The sequences were then analysed by using ChromasPro (version 1.5) software (http://technelysium.com.au/wp/chromaspro/) and Bio Edit alignment editor (version 7.2) (https://bioedit.software.informer.com/7.2/). The sequenced samples were validated by BlastN (https://blast.ncbi.nlm.nih.gov/Blast.cgi) and *pvcsp* repeat types (VK210/VK247) was analysed by BLAST. The alignment of top hit resulted sequences were done by ClustalW (https://www.genome.jp/tools-bin/clustalw). The number of haplotypes (H), nucleotide diversity (π) and haplotype diversity (Hd) were calculated by DNAsp v5 [33]. The evolutionary relationships of the both genes were established, and evolutionary tree were constructed by the neighbour-joining method using Molecular Evolutionary Genetics Analysis (MEGA 7.0) software [34]. The neutral theory of natural selection and population bottlenecks were also measured by Tajima’s D, Fu and Li’s D*, and Fu and Li’s F* tests using DNAsp v5 [33]. The negative value of Tajima’s D, Fu and Li’s D* indicates an excess of rare alleles that might results from selective sweep. A plot was constructed to look for polymorphic patterns of the N- and C-terminal in Pakistani *pvmsp-1* gene by using the WebLogo program (https://weblogo.berkeley.edu/logo.cgi). The nucleotide sequences of *pvcsp* and *pvmsp-1* genes obtained from this study were submitted in NCBI database under accession number MT222296 to MT222330 and MT303819 to MT303848, respectively.

## Results

In the present study, a total of 150 blood samples were collected from *P. vivax* malaria infected patients from the hospitals of twin cities of Islamabad and Rawalpindi. The blood samples were verified by microscopic examination to ensure the *P. vivax* parasites and excluded the samples for presence of mixed *Plasmodium* species infections. The PCR product of 1100 bp for *pvcsp* gene and ~ 400 bp for partial sequence of block 2 for *pvmsp-1* gene.

### Sequence analysis of *pvcsp* gene

**Fig. 2:**
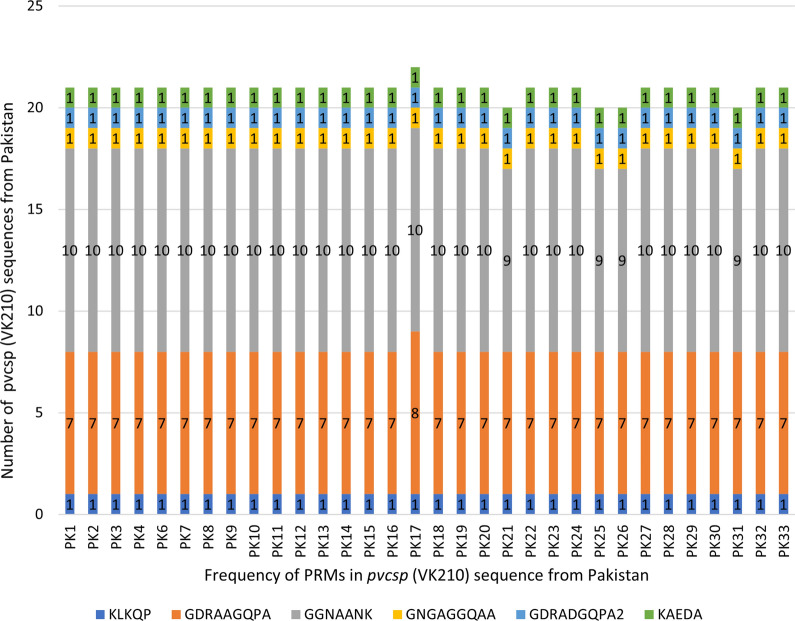
Frequency of peptide repeat motifs (PRMs) in the central repeat region (CRR) of Pakistani *pvcsp* (VK210) sequences

Multiple sequence alignment of the translated nucleotide sequences was carried out for the analysis of polymorphisms in the pre-, post- and central repeats of the *pvcsp* gene. The top hits for *pvcsp* gene were extracted from GenBank protein database using Blastp and one of the sequences of *pvcsp* gene of Iranian isolate was retrieved and used as reference sequence (KT588208.1). The multiple sequence alignment of extracted amino acid sequences was performed using ClustalW. When compared with the reference sequence (KT588208.1), the sequence analysis of *pvcsp* gene showed the VK210 and VK247 variant types infection. The *pvcsp* gene sequence analysis revealed that majority (92%; 32/35) of the *P. vivax* isolates were of VK210 variant and only 3 isolates were found to be VK247 type. All the *pvcsp* gene-based *P. vivax* variants started with the same pre-repeat sequence (KLKQP region I). In the central-repeat region (CRR), the VK210 sequences comprised of variable repeats of PRMs, GDRADGQPA (PRM1), GDRAAGQPA (PRM2) which were found in all the isolates. It was followed through two conserved post-repeat sequence GNGAGGQAA (PRM3) and GGNAANK (PRM4) and one post-repeat insert i.e., KAEDA region. The one-copy repeat region of GGNA was found after the CRR in all the analysed sequences. The frequency of peptide repeat motifs (PRMs) in the central repeat region (CRR) of *pvcsp* has been summarized in Fig. 2. The observed non-synonymous substitution based on diverse types of repetition in allotypes (RATs), which leads to different PRMs are mentioned in Table 1.

**Table 1:** Nucleotide sequence of four repeated allotypes (RATs) and the peptide repeat motif (PRMs) in the central-repeat region of *pvcsp* gene

PRMs	Nucleotide sequence of the repeat allotypes (RATs)
GDRADGQPA (PRM1)	GGAGACAGAGCAGATGGACAGCCAGCA GGAGACAGAGCAGATGGACAGCCAGCA GGTGATAGAGCAGCTGGACAACCAGCA GGTGATAGAGCAGATGGACAGCCAGCA GGCGATAGAGCAGCTGGACAGCCAGCA GGCGATAGAGCAGATGGACAGCCAGCA GGAGATAGAGCAGCTGGACAGCCAGCA GGCGATAGAGCAGATGGACAGCCAGCA
GDRAAGQPA (PRM2)	GGAGATAGAGCAGCTGGACAGCCAGCA GGCGATAGAGCAGATGGACAGCCAGCA GGAGATAGAGCAGCTGGACAACCAGCA GGTGATAGAGCAGCTGGACAACCAGCA GGAGATAGAGCAGATGGACAACCAGCA GGAGATAGAGCAGCTGGACAGCCAGCA GGAGATAGAGCAGCTGGACAGCCAGCA GGAGATAGAGCAGCTGGACAGCCAGCA
GNGAGGQAA (PRM3)	GGAGATAGAGCAGCTGGACAGCCAGCA
GGNAANK (PRM4)	GGAAATGGTGCAGGTGGACAGGCAGCA GGAGGAAATGCGGCAAACAAG

### *Pvcsp* CRR based genetic population structure

The population genetic structure based on the *pvcsp* CRR of the *P. vivax* isolates was analysed and compared with *pvcsp* isolates of neighboring countries Iran, India and Myanmar. The haplotype (gene) diversity of *pvcsp* was categorized into fifteen distinct haplotypes with an estimated Hd of 0.547 and ten distinct haplotypes with an estimated Hd of 0.345 in Pakistani and Iranian *pvcsp* samples respectively. The values for Tajima’s D, Fu and Li’s D* and F* tests are given in Table 2 for the *pvcsp* variants from Pakistan, Iran, India and Myanmar. The Fu and Li’s D* and F* values for CRR region was also positive suggested that the CRR region of *pvcsp* population of Pakistan was under positive natural selection. The nucleotide diversity in *pvcsp* population of Pakistan, Iran, India and Myanmar were highly significant (*P* < 0.05) as compared to haplotype diversity which was significant (*P* < 0.05) in Pakistan and Iran and non-significant (*P* > 0.05) in India and Myanmar. The *pvcsp* population from India and Iran showed high nucleotide diversity but values from Myanmar of *pvcsp* population were negative, suggesting negative selection. The values of the Tajima’s D, Fu and Li’s D* and F* values for CRR region was also positive for *pvcsp* population of Iran and India as shown in Table 2.

**Table 2:** Estimates of nucleotide, haplotype-diversity and DNA sequence polymorphisms of CRR region of *P. vivax pvcsp* and block 2 of *pvmsp-1* genes in Pakistan

Population	Gene analysed	No. of sequences	Fragment studied	Size of the fragment	No. of Haplotypes	Diversity ± SD	Fu & Li’s D*	Fu & Li’s F*	Tajima’s D*
				(codons)#		Nucleotide (π) Haplotype			
Pakistan	*pvcsp*	35	CRR	364	15	0.02371 ± 0.00056^†^ 0.084 ± 0.00701^†^	1.1787	1.12083	0.54276
Iran	*pvcsp*	28	CRR	360	10	0.02001 ± 0.00031^†^ 0.057 ± 0.00478^†^	1.0198	1.80767	0.43556
Myanmar	*pvcsp*	15	CRR	354	7	0.01781 ± 0.0008† 0.056 ± 0.015	− 1.20965	− 1.06781	− 0.78645
India	*pvcsp*	25	CRR	324	17	0.0370 ± 0.0064† 0.681 ± 0.076	1.05422	1.02237	0.35674
Pakistan	*pvmsp-1*	30	block 2	143	10	0.00162 ± 0.0000026^†^ 0.012 ± 0.00014^†^	1.86276	2.13897	1.6779
Iran	*pvmsp-1*	32	block 2	151	10	0.00159 ± 0.0000023^†^ 0.012 ± 0.00014^†^	1.78902	1.90878	1.56845
India	*pvmsp-1*	25	block 2	155	7	0.0212 ± 0.0005† 0.989 ± 0.010	1.55433	1.744009	1.66792

^†^ *P* < *0.05*

^#^ codon

### Phylogenetic analysis of *pvcsp* gene

**Fig. 3:**
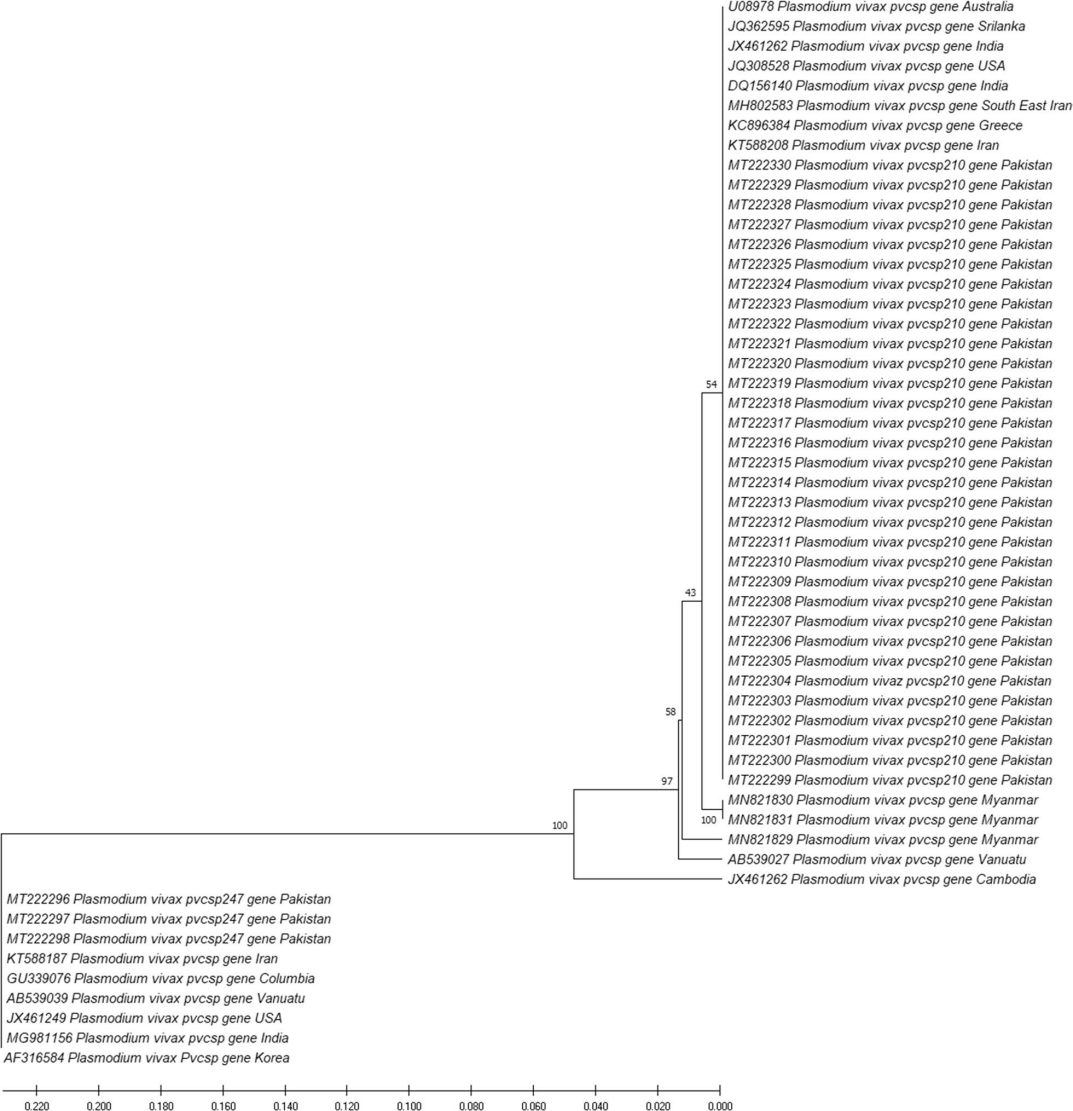
Phylogenetic relationships of VK210 and VK247 (based on *pvcsp* gene analysis) isolates from Pakistan. The Neighbour-Joining method was used to deduce evolutionary history with the branch length of = 0.50423062

A phylogenetic tree drawn from the sequence findings of *pvcsp* gene is presented in Fig. 3. Two separate clades can be inferred from the tree; one having VK210 variant type while the other has VK247 variant type of *pvcsp* gene. Four sub-clusters of VK210 and VK247 can be distinguished in the leading clade. The associated taxa were clustered together and shown after the branches with the branch length as of the evolutionary distances used to calculate the phylogenetic tree. The evolutionary distance was computed by the p-distance method and analysed using 55 nucleotide sequences. All the positions that had gaps or missing data were discarded. VK210 strain sequences from Pakistan showed 54% identity with *pvcsp* sequences from countries such as Iran, Greece, India, USA, Sri Lanka, Australia, Vanuatu and 100% with Myanmar, whereas the sequences of VK247 strains from Pakistan showed 100% identity with *pvcsp* sequences from Iran, Columbia, Vanuatu, USA, India and Korea.

### Sequence analysis of *pvmsp-1* gene

**Fig. 4:**
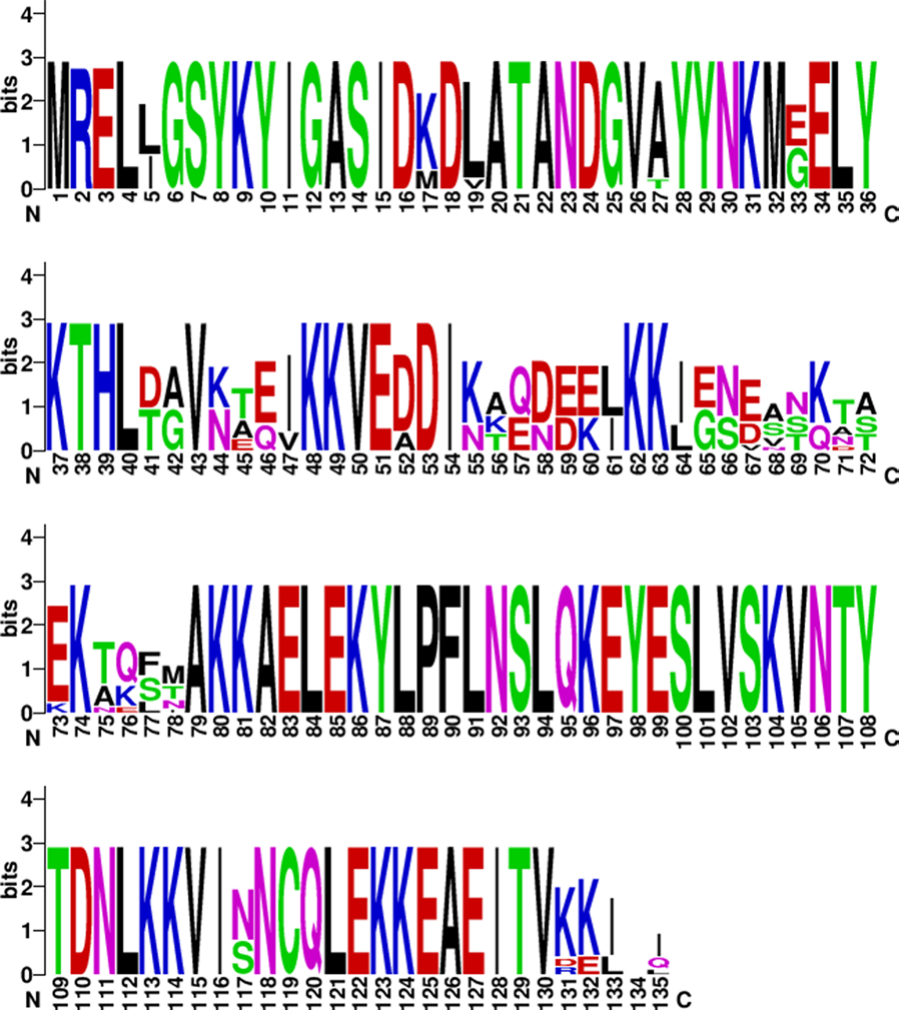
Analysis of polymorphic patterns of Pakistani *pvmsp-1* gene

The top hits for *pvmsp-1* gene were extracted from GenBank protein database using Blastp and one of the sequences of *pvmsp-1* gene of Iranian isolate was retrieved and used as reference sequence (KX697612.1). Sequence of *pvmsp-1* gene was compared with reference sequence KX697612.1 of Iranian *P. vivax* strain. It revealed that *pvmsp-1* gene sequences of 30 samples were corresponding to partial sequence of block 2 of *pvmsp-1* gene. Overall, 13 single nucleotide polymorphisms (SNPs) were found amongst 30 sequences with an average π value of 0.00143 in block 2 of *pvmsp-1* gene. The average conserved sequence between Pakistani and reference Iranian *pvmsp-1* gene was C: 0.835 indicating that sequences have remained relatively unchanged with close evolutionary relationship. Overall genetic polymorphisms of the *pvmsp-1* population were analyzed as shown in Fig. 4. The low frequencies of uneven amino acid changes were identified at block 2 of the *pvmsp-1* gene. The significant variation was observed from amino acid position K55N to M78T/N showing uneven and low frequencies with less conserved sequence of *pvmsp-1.*

### *Pvmsp-1* N-terminal based genetic population structure

Population genetic structure based on the N-terminal of *pvmsp-1* gene of the *P. vivax* isolates was analysed and compared with isolates of neighboring country Iran as shown in Table 2. The haplotype diversity of *pvmsp-1* gene was comparable between two countries ranging from 0.012 to 0.989. Adding to this, Tajima’s D, Fu and Li’s D* and F* tests also accepted occurrence of a neutral model of polymorphism with values for Fu and Li’s D* and Fu and Li’s F* are given in Table 2 for the *pvmsp-1* variants from Pakistan and Iran. The Fu and Li’s D* and F* values for *pvmsp-1* population were also positive. The results of *pvmsp-1* population of Pakistan also indicated that positive natural selection may occur in the region. The overall nucleotide and haplotype diversity were 0.00162 ± 0.0000026 and 0.012 ± 0.00014 respectively. The nucleotide diversity in *pvmsp-1* population of Pakistan, Iran and India were highly significant (*P* < 0.05) as compared to haplotype diversity, which was significant (*P* < 0.05) in Pakistan and Iran. The effect of natural selection was estimated by the Tajima’s D which was 1.67790 (*P* > 0.10).

### Phylogenetic analysis of *pvmsp-1* gene

**Fig. 5:**
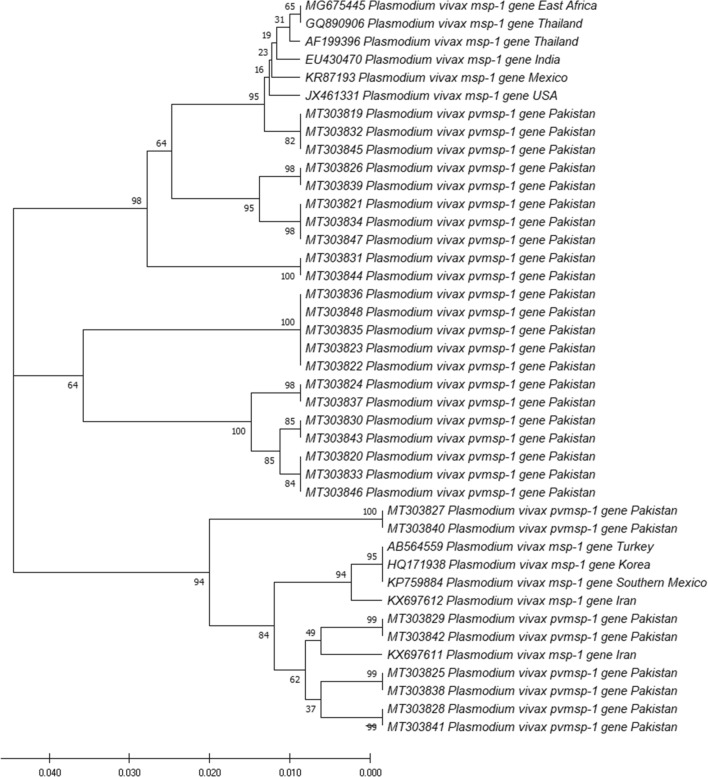
Phylogenetic relationships (based on *pvmsp-1* gene) of *P. vivax* isolates from Pakistan. The Neighbour-Joining method was used to deduce evolutionary history with the branch length of = 0.21993778

Based on sequence of *pvmsp-1* gene, a phylogenetic tree was constructed (Fig. 5). Two distinct clades can be inferred from the tree. The first is divided further into three sub-clades and contains Pakistani isolates and isolates belong to East Africa, Thailand, Mexico, India and USA with these isolates having 16 to 95% identity with sequences from Pakistani *pvmsp-1* population. Second clade is further divided into two sub-clades having Turkey, Iran, Korea and Southern Mexico isolates in addition to sequences of Pakistani isolates with 62–94% sequence identity.

## Discussion

Malaria is one of the major public health concerns with limited data in Pakistan [35]. As Pakistan shares border with malaria endemic countries like Iran, India and Afghanistan, the human migration across the border is inevitable possibly facilitating substantial cross-border transmission of malaria. The resultant recombination leads to genetic diversity and affects the frequency of new alleles in the parasite population [36, 37]. There are limited studies from Pakistan which have analysed the diversity of local *P. vivax* in detail [5, 6, 24, 36]. *pvcsp* and *pvmsp-1* are among the other important genetic markers used by the researchers to understand population structure and evolutionary dynamics from different geographical regions [5, 26]. In the present study, the genetic polymorphism in the *pvcsp* and *pvmsp-1* genes were studied in detail. The results of the study support the findings of the previously published data showing that VK210 strain is predominant type with the prevalence rate ranging between 56–100% in Iran [20, 24], Myanmar [15, 40], Brazil [39], India [14, 49], Thailand [41, 42], Azerbaijan [19], China [13, 42] and Mexico [43]. There are only few malaria endemic areas where VK247 isolates are commonly present [39].

The analysis of translated nucleotide sequences suggested that GDRADGQPA (PRM1) and GDRAAGQPA (PRM2) are two major PRMs. Earlier studies also reported the dominance of the two prime PRMs in the clinical isolates [8, 9, 14]. All these isolates were composed of similar pre-repeat sequence (KLKQP) region and conserved post-repeat sequence GGNAANK (PRM4) present as a last section in all of the VK210 isolates as aforementioned in studies from India, Iran and Sri Lanka [9, 14, 20]. Another peptide repeat motif GNGAGGQAA (PRM3) was found at lower frequency (0.6%) in the isolates. The variations exist in the amino acid and nucleotide sequences of the *Plasmodium* antigens due to variations in the repeat unit numbers which is indicative of the natural selection pressure by the host immune system [14, 44]. The arrangement of the main PRM1 and PRM2 factors leads to 15 different haplotypes of *pvcsp.* The analysis of CRR region of Pakistani *pvcsp* isolates is indicative of positive selection when compared with *pvcsp* isolates of Iran and India. The negative Tajima’s D values of Myanmar *pvcsp* population imply purifying negative selection [15]. The evidence from the previous studies has reported that the arrangements and numbers of PRMs in CRR are indicative of occurrence of phenomenon of natural selection on the *pvcsp* isolates [15, 45, 46].

The *pvmsp-1* is one of the most promising vaccine candidates and is available for antigenic and genetic variation studies of *P. vivax* populations [47]. In this study, partial sequence (~ 400 bp) at block 2 of *pvmsp-1* gene has depicted a high-level of diversity, which is in concordance with what has already been observed in neighboring country Iran [24], and also in previous study from Pakistan [5]. In the northwestern region of Thailand, a high degree of mutational variety was observed in *pvmsp-1* genes of *P. vivax* isolates [29, 46]. The *pvmsp-1* population of Pakistan has also indicated positive natural selection when compared with *pvmsp-1* isolates of Iran and India. The significantly positive values of Fu and Li’s D* and F* tests may be the result of balancing the selection and population bottlenecks. The sequence diversity of the population is best studied by the intragenic recombination of *pvmsp-1* gene where the allelic recombination frequencies may aid as a character reference for understanding the parasitic population structure [28]. Kibria et al*.* [26] also indicated a high genetic diversity of *pvmsp-1* gene which undergoes selective pressure for the existence and spread of the parasite. Similar pattern of genetic diversity was observed in *Pfmsp-1* gene of *P. falciparum* populations in Pakistan [36]. The *pvmsp-1* gene sequences were useful in distinguishing the two central localities of origin in terms of geography as well as helping them to group in two different clades. These biological groups are further subdivided into different clusters according to their geographic origin [47]. The polymorphic nature of MSP-1 markers can be used to differentiate between reemergence and reinfections and to determine ultimate shifts in population dynamics of parasites [48].

Other studies carried out in India and Brazil have suggested that the mode of evolution in *pvcsp* gene can lead to cohort of variants that can elude the host immune response under the effect of both mitotic recombination and positive selection of new variants of *P. vivax* [14, 17, 49]. Therefore, it is safe to assume that the wide variety of *P. vivax* may be interrelated with multiple other variables including, but not limited to, genetic and biological characteristics, immunity of the host and the displacement of individuals within the boundaries of the endemic areas. Furthermore, the spread of *P. vivax* infections is also reinforced by relapse and early gametocytaemia, which in turn sustains local diversity, paving way for a more efficient transmission to the vector mosquitoes [13, 50]. The results revealed a broad range of genetic variety of *pvcsp* and *pvmsp-1* genes in *P. vivax* population. The localities of the study areas from where samples were collected are inhabited by various ethnicities which suggested that the migration of people may carry diverse parasite entities that increase the variety of the gene-pool. The individuals infected by the disease might carry various clones which may recombine during the sexual stage of the mosquito leading to the production of offspring with new *pvcsp* genotypes. This prevalent phenomenon could strengthen the introduction of new strains of *P. vivax* into the regions where conditions for malaria transmission are conducive [13, 26].

## Conclusion

The *pvcsp* and *pvmsp-1* genes have been extensively studies to understand the genetic diversity of *P. vivax* population globally. This study highlights the genetic characterization of *P. vivax* isolates from Pakistan and results revealed the polymorphism and genetic diversity especially at the CRR of *pvcsp* and block 2 of *pvmsp-1* genes, respectively. The results specify the effect of natural selection pressure on the malaria parasite for its survival and transmission. The base-line data presented here warrants further studies to investigate more into the genetic diversity of *P. vivax* with large sample size from across the country. It will help in better understanding of population dynamics of *P. vivax* as well as unstable reemergence of the infection due to population dynamics of parasite that will help to control malaria at individual and community level.

## Data Availability

The datasets used and/or analysed during the current study are available from the corresponding author on reasonable request.
